# Association of Vitamin K Insufficiency as Evaluated by Serum Undercarboxylated Osteocalcin With Frailty in Community-Dwelling Older Adults

**DOI:** 10.3389/fragi.2022.865178

**Published:** 2022-04-13

**Authors:** Kotaro Azuma, Yosuke Osuka, Narumi Kojima, Hiroyuki Sasai, Hunkyung Kim, Satoshi Inoue

**Affiliations:** ^1^ Department of Systems Aging Science and Medicine, Tokyo Metropolitan Institute of Gerontology, Tokyo, Japan; ^2^ Research Team for Promoting Independence and Mental Health, Tokyo Metropolitan Institute of Gerontology, Tokyo, Japan

**Keywords:** vitamin K, undercarboxylated osteocalcin (ucOC), osteocalcin (OC), frailty, cross-sectional study

## Abstract

Frailty is the state of having a reduced ability to recover from stress. Intervention in frailty is important for fulfilling healthy longevity. Vitamin K is a fat-soluble vitamin contained in vegetables and fermented foods. Although vitamin K is shown to be associated with several age-related diseases, studies on the association of vitamin K intake and frailty in the elderly population are limited. In the present study, a total of 800 community-dwelling older adults (mean age = 75.9) were recruited for a comprehensive geriatric health examination, including frailty evaluation based on the Japanese version of the Cardiovascular Health Study criteria. Serum concentrations of total osteocalcin (OC) and undercarboxylated osteocalcin (ucOC) were measured. The ratio of ucOC and OC (ucOC/OC), which reflects vitamin K insufficiency, was calculated for each participant, and the values were divided into quartiles. A binary logistic regression analysis was performed to evaluate the risk of frailty for each quartile of ucOC/OC, with the lowest quartile as the reference. Significant association of frailty and the highest quartile of ucOC/OC was found with the odds ratio of 2.49 (*p* = 0.023) with adjustment with age, sex, body mass index, dietary intake, and several clinical characteristics. When the analysis was repeated in each component of frailty, the highest quartiles of ucOC/OC had the tendency of association with “slow walking speed” and “low activity.” Our findings demonstrated the association between vitamin K insufficiency and frailty in the elderly population. Our analysis also suggests that vitamin K insufficiency could be associated with selected components of frailty.

## Introduction

Vitamin K is a fat-soluble vitamin contained in vegetables and fermented foods (Suttie and Booth, 2011; Fu et al., 2017; Tarvainen et al., 2019). Vitamin K contained in foods is classified into two forms, namely, vitamin K1 (phylloquinone) and K2 (menaquinone). Vitamin K1 is abundant in vegetables (Suttie and Booth, 2011), whereas vitamin K2 is synthesized by microorganisms and is contained in fermented foods such as fermented soybeans (called “natto” in Japanese) (Fu et al., 2017; Tarvainen et al., 2019). Vitamin K was originally discovered as an essential factor for blood coagulation (Dam, 1935). Classically, the functions of vitamin K are explained by its role as an essential co-factor for the post-transcriptional modification of the proteins which is catalyzed by *γ*-glutamyl carboxylase (GGCX) (Nelsestuen et al., 1974; Stenflo et al., 1974). Substrates of GGCX include several coagulation factors (Nelsestuen et al., 1974; Stenflo et al., 1974; Bucher et al., 1976), matrix Gla protein (MGP) (Price et al., 1983), and osteocalcin (OC) (Price et al., 1976). To date, vitamin K is shown to function by other mechanisms, including transcriptional activation as a ligand for steroid and xenobiotic receptors (SXR) (Tabb et al., 2003; Azuma and Inoue, 2019). The latter function of vitamin K as an SXR ligand has been shown only in vitamin K2. Meanwhile, vitamin K1 can function as vitamin K2 since vitamin K1 is known to be converted to vitamin K2 by UbiA prenyltransferase domain–containing protein l (UBIAD1) in the mammalian body (Nakagawa et al., 2010).

Other than its clinical role in blood coagulation, vitamin K status is also associated with age-related diseases including osteoporosis (Booth et al., 2000; Kaneki et al., 2001; Yaegashi et al., 2008) and osteoarthritis (Neogi et al., 2006; Oka et al., 2009; Misra et al., 2013). By some clinical observational studies, vitamin K status was reported to be correlated with physical performance such as gait speed, hand grip strength, and mobility (Shea et al., 2016; van Ballegooijen et al., 2018; Shea et al., 2020; Smith et al., 2021), suggesting that vitamin K status may also be involved in sarcopenia. Sarcopenia is one of the underlying conditions of frailty, which is a state of reduced ability to recover from stress and is considered a reversible pre-disability stage. In older adults, conditions or diseases including falls and fractures, postoperative complications, disability, institutionalization, and mortality are shown to be outcomes of frailty (Fried et al., 2001).

Several epidemiological studies showed that frailty could be associated with the dietary pattern. Mediterranean diet was associated with the decreased risk of frailty (Kojima et al., 2018). Intake of fat-soluble vitamins such as vitamin A, D, and E was negatively associated with frailty prevalence (Hernández Morante et al., 2019; Pilleron et al., 2019). An observational study conducted in the Netherlands reported that vitamin K insufficiency evaluated by plasma dephospho-uncarboxylated matrix Gla protein (dp-ucMGP) was associated with a higher prevalence of frailty (Machado-Fragua et al., 2020). In the report, the dp-ucMGP of the population between 55 and 65 years old was analyzed (Machado-Fragua et al., 2020). As far as we know, the association of vitamin K status with frailty in the older adult population, nor the vitamin K status evaluated by undercarboxylated osteocalcin (ucOC) with frailty, has not been reported so far. We conducted a cross-sectional study in which the association of vitamin K status and frailty in a community-dwelling Japanese older adult population was examined. We utilized the ratio of the serum concentration of ucOC and total OC (ucOC/OC) as an indicator of vitamin K insufficiency. A higher value of ucOC/OC indicates a vitamin K insufficient state, which was validated with the actual vitamin K concentration in a previous study (Tsugawa et al., 2006).

## Materials and Methods

### Study Design

In 2020, a total of 800 people (mean age = 75.9) were recruited as a follow-up study of the baseline examinations conducted from 2017 to 2019 by mailing community-dwelling older adults randomly from the Basic Resident Register of the Itabashi district in metropolitan Tokyo. Among them, 88.8% (710 people) were females. They were invited to a comprehensive geriatric health examination, including a frailty evaluation and blood test. Information on past medical history and dietary intake is obtained by using a self-reporting questionnaire. Grip strength and walking speed were measured by the staff of the Tokyo Metropolitan Institute of Gerontology. For the measurement of walking speed, participants were asked to walk for 11 m, and the walking time required for the 5 m in the middle of the way was then measured. Frailty was evaluated by the Japanese version of the Cardiovascular Health Study criteria (Satake et al., 2017). The five components of frailty, namely, “shrinking,” “weakness,” “exhaustion,” “slowness,” and “low activity,” were evaluated. People having more than two components were judged as being frail. “Shrinking” was defined as unintentional body weight loss equal to or more than 3 kg in the past 6 months. “Weakness” was defined as having a hand-grip strength of less than 26 kg (male) or 18 kg (female). “Exhaustion” was judged as positive when the participants answered “Yes” to the question, “Do you feel tired without a reason for the past 2 weeks?” “Slowness” was defined as a walking speed of less than 1.0 m/s. “Low activity” was judged positive when the participants answered “No” to both the questions, “Do you engage in moderate levels of physical exercise or sports aimed at health?” and “Do you engage in low levels of physical exercise aimed at health?” Serum OC and ucOC concentration were measured by BML Inc. (Tokyo, Japan). This study was approved by the institutional ethical committee of the Tokyo Metropolitan Institute of Gerontology (approval number: H18-17, R1-20). All participants provided written informed consent.

### Statistical Analyses

The serum ucOC/OC values are divided into quartiles (Q1, Q2, Q3, and Q4). Descriptive statistics were used to compare patients’ characteristics according to the levels of ucOC/OC using one-way analysis of variance for continuous variables and a chi-square test for categorical variables. Binary logistic regression analysis was performed to evaluate the association of frailty with the following variables: age (continuous), sex (binary), body mass index (categorical; <18.5, 18.5–25, 25–30, and 30+), hypertension (binary), stroke (binary), heart disease (binary), diabetes (binary), dyslipidemia (binary), osteoporosis (binary), smoking status (binary, both current smokers and ex-smokers are defined as having positive smoking status), ucOC/OC (categorical), and dietary intake (categorical). Components of dietary intake used for the analysis include meat, fish, eggs, soybean products, milk, cheese, potatoes, vegetables, fruits, seaweeds, and “oils and fats.” Vegetables are specified to be dark-colored vegetables, including carrots, spinach, pumpkins, and tomatoes. “Oils and fats” indicate stir-fried foods, butter, and margarine. Dietary intake was positive when the frequency of intake was nearly every day. In the chi-square test, the specific intake of “fermented soybeans,” one of the soybean products, was included in the analysis. In the case of fermented soybeans, the frequency of intake of more than 3 days a week was defined as positive. Binary logistic regression analysis was repeated to evaluate the association of each component of frailty (shrinking, weakness, exhaustion, slowness, and low activity) with the same variables in the analysis of frailty itself. IBM SPSS Statistics version 25 software (IBM Corporation, Armonk, NY, United States) was used for all statistical analyses.

## Results

The characteristics of the study population according to the levels of serum ucOC/OC divided into quartiles are summarized in Table 1. Among the participants, 55 (6.9%) were frail according to the criteria. The frequent intake of fermented soybeans showed a significantly negative correlation with a high value of ucOC/OC (*p* < 0.001 in chi square test). Since the fermented soybeans contain a high amount of vitamin K2 (Tarvainen et al., 2019), this result can be regarded as one of the validations that ucOC/OC is serving as an indicator of vitamin K insufficiency in our study. Meanwhile, ucOC/OC was not associated with the intake of soybean products as a whole, which contains other soybean products including “miso” and “tofu” together with fermented soybeans. The association of ucOC/OC and the intake of vegetables, which are a known source of vitamin K1, were not detected, either.

**TABLE 1:** Characteristics of the study populations according to the levels of ucOC/OC^a^.

Characteristic	ucOC/OC				*p* value^b^	Total
	Q1	Q2	Q3	Q4		
ucOC/OC	<0.1627	0.1627–0.1942	0.1942–0.2324	>0.2324		
N	200	200	200	200		800
Age (year)	76.5 ± 4.8	75.9 ± 4.7	75.8 ± 4.7	75.5 ± 5.3	0.193	75.9 ± 4.9
Female	170 (85.0%)	173 (86.5%)	185 (92.5%)	182 (91.0%)	*0.054*	710 (88.8%)
BMI	22.8 ± 3.2	23.1 ± 3.3	32.4 ± 3.1	23.3 ± 3.6	0.297	23.2 ± 3.3
Hypertension	90 (45.0%)	69 (34.5%)	91 (45.5%)	91 (45.5%)	*0.065*	341 (42.6%)
Stroking status	2 (1.0%)	1 (0.5%)	7 (3.5%)	10 (5.0%)	***0.012***	20 (2.5%)
Heart disease	35 (17.5%)	25 (12.5%)	23 (11.5%)	28 (14.1%)	0.326	111 (13.9%)
Diabetes	37 (18.6%)	21 (10.5%)	27 (13.5%)	18 (9.0%)	***0.023***	103 (12.9%)
Dyslipidemia	100 (50.5%)	94 (47.0%)	92 (46.2%)	94 (47.0%)	0.831	380 (47.5%)
Osteoporosis	79 (40.1%)	55 (27.6%)	44 (22.1%)	41 (20.6%)	***< 0.001***	219 (27.4%)
Smoking status^c^	59 (29.5%)	41 (20.5%)	42 (21.0%)	49 (24.5%)	0.128	191 (23.9%)
Frailty	15 (7.5%)	10 (5.0%)	8 (4.0%)	22 (11.0%)	***0.028***	55 (6.9%)
Dietary intake^d^						
Meat	77 (38.5%)	74 (37.0%)	57 (28.5%)	68 (34.0%)	0.159	276 (34.5%)
Fish	67 (33.5%)	65 (32.5%)	71 (35.5%)	70 (35.0%)	0.918	273 (34.1%)
Eggs	91 (45.5%)	84 (42.0%)	66 (33.0%)	85 (42.5%)	*0.065*	326 (40.8%)
Soybean products	103 (51.5%)	101 (50.5%)	104 (52.0%)	98 (49.0%)	0.936	406 (50.8%)
Fermented soybeans^e^	112 (56.0%)	107 (53.5%)	91 (45.5%)	63 (31.5%)	***< 0.001***	373 (46.6%)
Milk	109 (54.5%)	119 (59.5%)	104 (52.0%)	104 (52.0%)	0.388	436 (54.5%)
Cheese	36 (18.0%)	47 (23.6%)	29 (14.5%)	38 (19.0%)	0.138	150 (18.8%)
Potatoes	21 (10.5%)	13 (6.5%)	15 (7.5%)	24 (12.0%)	0.191	73 (9.1%)
Vegetables	138 (69.0%)	129 (64.5%)	124 (62.0%)	127 (63.5%)	0.496	518 (64.8%)
Fruits	119 (59.5%)	114 (57.0%)	113 (56.5%)	113 (56.5%)	0.918	459 (57.4%)
Seaweeds	37 (18.5%)	43 (21.5%)	38 (19%)	43 (21.5%)	0.812	161 (20.1%)
Oils and fats	114 (57.0%)	116 (58.3%)	92 (46.2%)	106 (53.0%)	*0.070*	428 (53.6%)

a Values are means ± SDs, for continuous variables or frequency (%) for categorical variables.

b *p* values are for one-way analysis of variance (continuous variables) and the chi-square test (categorical variables).

c Current smokers and ex-smokers are included.

d Numbers and ratios of the participants who consume the indicated food or drink nearly every day are shown, except for the intake of fermented soybeans.

e Numbers and ratios of the participants who consume fermented soybeans more than 3 days a week are shown.

Significant *p* values (*p* < 0.05) are expressed in bold italics, and *p* values less than 0.1 (0.05 ≤ *p* < 0.1) are expressed in italics.

Abbreviations: BMI, body mass index; OC, osteocalcin; ucOC, undercarboxylated osteocalcin.

Next, binary logistic regression analysis was performed to determine the association between frailty and each characteristic, including age, sex, body mass index, hypertension, stroke, heart disease, diabetes, dyslipidemia, osteoporosis, smoking status, dietary intake, and ucOC/OC. Among them, age, hypertension, diabetes, osteoporosis, and the highest quartile (Q4) of ucOC/OC with the lowest quartile (Q1) as the reference were significantly associated with frailty (Table 2). The odds ratio (OR) of the highest quartile (Q4) of ucOC was 2.49, and its 95% confidence interval (CI) was from 1.13 to 5.45 (*p* = 0.023). These results indicated that older age, hypertension, diabetes, osteoporosis, and vitamin K insufficiency are associated with frailty. In addition, the body mass index between 25 and 30 and the positive smoking status tended to correlate with frailty (Table 2).

**TABLE 2:** Association of characteristics with frailty.

Characteristic	*B*	OR (95% CI)	*p* value
Age	0.108	1.12 (1.04–1.19)	***0.001***
Sex (women)	0.362	1.44 (0.50–4.11)	0.499
BMI			
<18.5	0.497	1.64 (0.50–5.42)	0.415
18.5–25		Ref	
25–30	0.580	1.79 (0.93–3.44)	*0.082*
30 <	0.695	2.00 (0.40–9.93)	0.395
Hypertension	0.637	1.89 (1.00–3.56)	***0.049***
Stroke	0.297	1.35 (0.34–5.37)	0.674
Heart disease	0.171	1.19 (0.56–2.52)	0.656
Diabetes	0.818	2.27 (1.08–4.74)	***0.030***
Dyslipidemia	0.081	1.08 (0.57–2.05)	0.804
Osteoporosis	0.912	2.49 (1.27–4.89)	***0.008***
Smoking status	0.678	1.97 (0.93–4.17)	*0.077*
ucOC/OC			
Q1		Ref	
Q2	0.129	1.14 (0.46–2.81)	0.780
Q3	−0.304	0.74 (0.28–1.94)	0.538
Q4	0.910	2.49 (1.13–5.45)	***0.023***
Dietary intake ^a^			
Meat	−0.363	0.70 (0.33–1.45)	0.332
Fish	−0.062	0.94 (0.45–1.95)	0.868
Eggs	0.341	1.41 (0.75–2.65)	0.293
Soybean products	0.051	1.05 (0.54–2.05)	0.882
Milk	0.182	1.20 (0.64–2.26)	0.573
Cheese	−0.477	0.62 (0.26–1.47)	0.279
Potatoes	−0.004	1.00 (0.37–2.70)	0.993
Vegetables	0.584	1.79 (0.88–3.68)	0.111
Fruits	−0.182	0.83 (0.44–1.57)	0.573
Seaweeds	0.082	1.09 (0.50–2.38)	0.839
Oils and fats	0.077	1.08 (0.57–2.05)	0.814

a Intake of indicated food or drink every day is considered positive.

Binary logistic regression analysis was performed to evaluate the association of frailty with the following variables: age (continuous), sex (binary), BMI (categorical), hypertension (binary), stroke (binary), heart disease (binary), diabetes (binary), dyslipidemia (binary), osteoporosis (binary), smoking status (binary), ucOC/OC (categorical), and dietary intake (categorical).

Abbreviations: B, logistic regression coefficient; BMI, body mass index; CI, confidence interval; OC, osteocalcin; OR, odds ratio; Ref, reference; ucOC, undercarboxylated osteocalcin.

Significant *p* values (*p* < 0.05) are expressed in bold italics, and *p* values less than 0.1 (0.05 ≤ *p* < 0.1) are expressed in italics.

**FIGURE 1:**
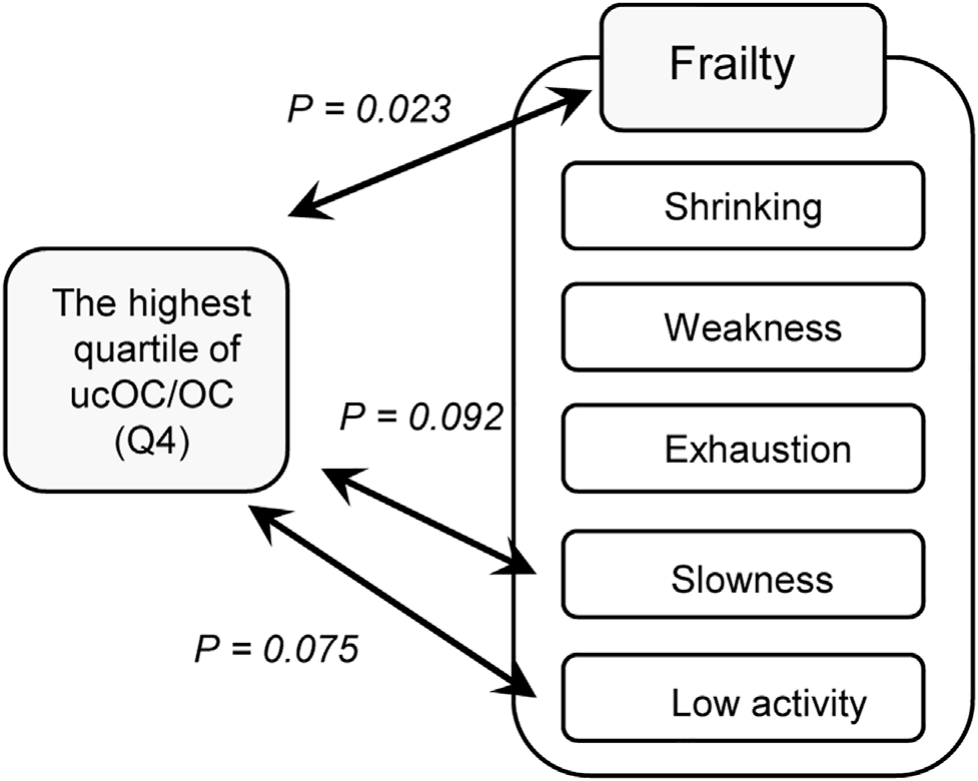
Association of vitamin K insufficiency and frailty. A diagram representing the results of the present study is shown. Vitamin K insufficiency was evaluated by the ratio of undercarboxylated osteocalcin and osteocalcin (ucOC/OC) The highest quartile of ucOC/OC, which reflects relatively weak vitamin K function *in vivo,* was significantly associated with frailty as determined by the Japanese version of the Cardiovascular Health Study criteria (*p* = 0.029), as shown in Table 2. Among the components of frailty in the criteria, the highest quartile of ucOC/OC tended to be associated with “slowness” (*p* = 0.092) and “low activity” (*p* = 0.075), as shown in Table 3.

Frailty is composed of five components, including shrinking (weigh loss of 2 or more kg in the past 6 months), weakness (grip strength of less than 26 kg in men or less than 18 kg in women), exhaustion (feeling of tiredness without a reason in the past 2 weeks), slowness (walking speed of less than 1.0 m/s), and low activity (no engagement in physical exercise aimed at health). When the analysis was repeated with each component of frailty, the highest quartile of ucOC/OC had a tendency of association with “slowness” (OR 1.80, 95% CI 0.91 to 3.56, and *p* value 0.092) and “low activity” (OR 1.64, 95% CI 0.95 to 2.83, and *p* value 0.075) with adjustment for age, sex, body mass index, hypertension, stroke, heart disease, diabetes, dyslipidemia, osteoporosis, smoking status, and dietary intake (Table 3). The results of our study are summarized in Figure 1.

**TABLE 3:** Associations between ucOC/OC and components of frailty.

	ucOC/OC			
	Q1	Q2	Q3	Q4
N	200	200	200	200
Components of frailty				
Shrinking				
OR (95% CI)	Ref	0.81 (0.43–1.51)	1.00 (0.55–1.82)	1.24 (0.70–2.22)
*p* value		0.502	0.991	0.465
Weakness				
OR (95% CI)	Ref	1.27 (0.76–2.11)	1.02 (0.61–1.72)	1.38 (0.83–2.30)
*p* value		0.361	0.932	0.220
Exhaustion				
OR (95% CI)	Ref	1.02 (0.63–1.67)	0.73 (0.44–1.22)	1.12 (0.69–1.82)
*p* value		0.924	0.235	0.645
Slowness				
OR (95% CI)	Ref	0.73 (0.33–1.62)	0.90 (0.42–1.90)	1.80 (0.91–3.56)
*p* value		0.440	0.775	*0.092*
Low activity				
OR (95% CI)	Ref	0.82 (0.45–1.49)	1.45 (0.83–2.53)	1.64 (0.95–2.83)
*p* value		0.507	0.193	*0.075*

A binary logistic regression analysis was performed to evaluate the association of components of frailty with ucOC/OC (categorical). The data were adjusted for the following variables: age (continuous), sex (binary), hypertension (binary), stroke (binary), heart disease (binary), diabetes (binary), dyslipidemia (binary), osteoporosis (binary), and smoking status (binary).

*p* values less than 0.1 (0.05 ≤ *p* < 0.1) are expressed in italics.

Abbreviations: CI, confidence interval; OC, osteocalcin; OR, odds ratio; Ref, reference; ucOC, undercarboxylated osteocalcin.

## Discussion

In the present study, we demonstrated the association of vitamin K insufficiency as evaluated by the serum ucOC/OC ratio with frailty in a community-dwelling older adult population. Furthermore, our analysis suggests that vitamin K insufficiency tended to be associated with slow walking speed and low activity.

In the epidemiological studies evaluating vitamin K insufficiency, vitamin K status has been estimated by food-frequency questionnaires (Booth et al., 2000; Oka et al., 2009), plasma concentration of dp-ucMGP (Shea et al., 2016; van Ballegooijen et al., 2018; Shea et al., 2020), ucOC/OC (Smith et al., 2021), or direct measurement of vitamin K concentration by high-performance liquid chromatography (HPLC) (Kaneki et al., 2001; Neogi et al., 2006; Misra et al., 2013; Shea et al., 2016; Shea et al., 2020). Those methods require relatively special methods and/or facilities, except for ucOC/OC, which may limit the translation of these findings into clinical settings. Since the measurements of serum ucOC and OC are simple and widely available, our study would inspire studies on diseases in which vitamin K insufficiency could be involved.

A possible association with vitamin K insufficiency and slow walking speed was in line with previous reports (Shea et al., 2016; van Ballegooijen et al., 2018). The reports on the mechanism of vitamin K action on physical performance are limited. A study using rat models showed that vitamin K increased slow-twitch muscle fibers and improved their mitochondrial function (Su et al., 2022), which may partially explain the effect of vitamin K on the muscular tissue. The effect of vitamin K on sphingolipid metabolism might be one of the underlying mechanisms. It was shown that ceramide accumulation was significantly correlated with decreased lean mass in the study of a small number of human subjects (Rivas et al., 2012), while sphingosine 1-phosphate had trophic action on denervated muscle in a rat model (Zanin et al., 2008). Since vitamin K is known to regulate sphingolipid metabolism (Lev and Milford, 1972) and warfarin treatment was shown to reduce certain types of sphingolipids in the mouse brain, including sulfatides, sphingomyelin, and cerebrosides (Sundaram and Lev, 1988), vitamin K may affect muscular performance, although the effect of vitamin K on ceramide or sphingosine 1-phosphate has not been studied yet to the best of our knowledge.

We proposed another possible association between vitamin K and “low activity.” The low activity can be caused by a sedentary lifestyle and decreased physical performance. It can be hypothesized that vitamin K may affect lifestyle by regulating psychological aspects. In an old study using vitamin K-deficient rats by administrating warfarin, hypoactivity and lack of exploratory behavior were observed (Cocchetto et al., 1985). A study using rat models demonstrated that vitamin K decreased depression-like behavior in the forced swim test and increased the score of the social interaction test (Gancheva and Zhelyazkova-Savova, 2016), which supported the possible effect of vitamin K on behavior. It is intriguing that osteocalcin was shown to be a hormone affecting behavior (Berger et al., 2019). In mice exposed to acute stress with trimethylthiazoline (fox odor), osteocalcin was released from osteoblasts and mediated the acute stress response by suppressing parasympathetic tone. In this function, ucOC is considered to be an active form, while carboxylated osteocalcin is considered to be an inactive form. In this sense, vitamin K insufficiency may be more likely to induce a fight or flight response and may transiently enhance physical performance. Meanwhile, it can be speculated that augmented stress response by vitamin K insufficiency leads to “low activity” in ordinary conditions.

We noticed that there are several limitations to this study. First, the majority of the population was females in our analysis. This problem could be overcome to some extent by using the statistical method of adjusting sex. Second, we did not have detailed information on the medications that potentially affected the function of vitamin K, such as anticoagulants. Therefore, our results may also include the effect of suppressive vitamin K function by anticoagulants on frailty. Third, we did not have information on the medication’s effect on bone turnover, which may affect the concentration of OC. Our analysis could be less susceptible to the absolute amount of OC by calculating the ratio of ucOC to OC. Since our study is an observational study, we could not discuss the cause-effect relationship between vitamin K and frailty. Considering the importance of intervention to frailty for fulfilling healthy longevity, we hope that our study could be one of the backgrounds to prompt clinical intervention studies clarifying the effect of vitamin K supplementation on the reversing frail state.

In summary, the present study demonstrated the association between vitamin K insufficiency and frailty in the older adult population. Our findings could be a clue to designing further studies on how vitamin K functions in frailty and its underlying conditions.

## Data Availability

The raw data supporting the conclusion of this article will be made available by the authors, without undue reservation.
